# ASK1 inhibition: a therapeutic strategy with multi-system benefits

**DOI:** 10.1007/s00109-020-01878-y

**Published:** 2020-02-14

**Authors:** Jacqueline M. Ogier, Bryony A. Nayagam, Paul J. Lockhart

**Affiliations:** 1 The Bruce Lefroy Centre, The Murdoch Children’s Research Institute, Parkville, Victoria Australia; 2 Department of Paediatrics, The University of Melbourne, Parkville, Victoria Australia; 3 Department of Audiology and Speech Pathology, The University of Melbourne, Parkville, Victoria Australia; 4 The Bionics Institute, East Melbourne, Victoria Australia

**Keywords:** Apoptosis signal-regulating kinase 1, MAP3K5, Clinical trial, ROS, MAPK, p38, JNK

## Abstract

p38 mitogen-activated protein kinases (P38α and β) and c-Jun N-terminal kinases (JNK1, 2, and 3) are key mediators of the cellular stress response. However, prolonged P38 and JNK signalling is associated with damaging inflammatory responses, reactive oxygen species–induced cell death, and fibrosis in multiple tissues, such as the kidney, liver, central nervous system, and cardiopulmonary systems. These responses are associated with many human diseases, including arthritis, dementia, and multiple organ dysfunctions. Attempts to prevent P38- and JNK-mediated disease using small molecule inhibitors of P38 or JNK have generally been unsuccessful. However, apoptosis signal-regulating kinase 1 (ASK1), an upstream regulator of P38 and JNK, has emerged as an alternative drug target for limiting P38- and JNK-mediated disease. Within this review, we compile the evidence that ASK1 mediates damaging cellular responses via prolonged P38 or JNK activation. We discuss the potential benefits of ASK1 inhibition as a therapeutic and summarise the studies that have tested the effects of ASK1 inhibition in cell and animal disease models, in addition to human clinical trials for a variety of disorders.

Mitogen-activated protein kinase kinase kinase 5 (MAP3K5), commonly known as apoptosis signal-regulating kinase 1 (ASK1), has emerged as a target for preventing p38 mitogen-activated protein kinase (MAPK14, 11, and 12/P38α, β, and γ) and c-Jun N-terminal kinase (MAPK8, 9, and 10/JNK1, 2, and 3)–mediated cell death and disease. Both P38 and JNK are associated with reactive oxygen species (ROS)–induced disease^1^, and numerous studies have demonstrated that P38 and JNK inhibition ameliorates cell death [3–7]. However, complete inhibition of P38 or JNK in vivo is problematic, given that these ubiquitously expressed proteins are also critical for cell survival and homeostatic and/or metabolic functions [8–11]. This is highlighted by the embryonic lethality of homozygous *P38*α and *Jnk1/2* knockout mice [12, 13]. In addition, homozygous *P38β* knockout mice exhibit defective skeletal development [14] and homozygous *Jnk1* knockout mice spontaneously develop intestinal tumours [15]. Negative outcomes have also been reported due to partial *P38* or *Jnk* expression, with heterozygous *P38*α knockout mice developing progressive renal dysfunction [16] and heterozygous *Jnk1* knockout mice exhibiting altered weight gain, hyperinsulinaemia, insulin resistance, inflammatory cytokine disruption, and reduced viability [17]. Serious side effects have also been observed when pharmacological inhibition of P38 or JNK is pursued in vivo [14, 18, 19]. For example, pamapimod, a P38 (α and β) inhibitor, did not significantly reduce joint swelling or improve mobility in individuals with rheumatoid arthritis in a phase II clinical trial. However, 35% of the participants receiving daily pamapimod (300 mg) experienced infection, 20% developed a skin rash, 15% became dizzy, and 13% had elevated hepatic enzymes indicative of liver damage [20].

The pro-survival or pro-death outcomes of P38/JNK activity are largely dependent on the duration of activation. Short-term activation is protective, inducing cellular repair mechanisms, whereas sustained P38/JNK phosphorylation initiates apoptotic and necrotic cell death cascades [21–26]. Therefore, an alternative therapeutic approach that targets a common, upstream molecule that is only activated by cell stress and capable of regulating both the P38 and JNK pathways is desirable. As an upstream converging point of cell stress signalling, ASK1 meets these criteria. Similar to P38 and JNK, ASK1 is ubiquitously expressed [27]. However, unlike P38 and JNK, ASK1 is primarily activated in response to cell stress (reviewed in Shiizaki et al. [28]). In particular, ASK1 activation is tightly controlled by redox signalling, due to the nature of its dithiol oxidoreductase binding partners, thioredoxin (TRX), glutaredoxin (GRX), and peroxiredoxin 1 (PRX1) [29–31] (Fig. 1). TRX, GRX, and PRX1 have redox active sites consisting of two cysteine residues that act as molecular switches [32]. When the cell is redox neutral, TRX, GRX, and PRX1 remain in a reduced state, bound to, and inactivating ASK1. TRX and PRX1 bind at the N-terminal domain of ASK1, and GRX at the C-terminus [30, 33–35]. Bound ASK1 is a substrate for ubiquitination and degradation [36]. Alternatively, cellular oxidative imbalance induces modifications of the cysteine sulphur atom of TRX, GRX, and PRX1. As a result, disulphide bonds form between the cysteine residues, inactivating TRX, PRX1, and GRX [37, 38]. Inactivated TRX, PRX1, and GRX dissociate from ASK1. Unbound ASK1 is then activated by auto-phosphorylation and a large multicomponent complex forms, referred to as the ASK1 signalosome [39]. This complex and associated ASK1 activity initiates the P38 and JNK signalling cascades [27, 40]. Importantly, ASK1 promotes the sustained and pro-apoptotic activation of P38 and JNK, without impacting short-term P38/JNK activity [41–43]. Therefore, ASK1 inhibition is unlikely to affect the pro-survival, homeostatic activities of P38 and JNK. This hypothesis is supported by the viability of *Ask1* knockout mice, which are healthy and long-lived, and show no developmental abnormalities [42, 44–48].

**Fig. 1:**
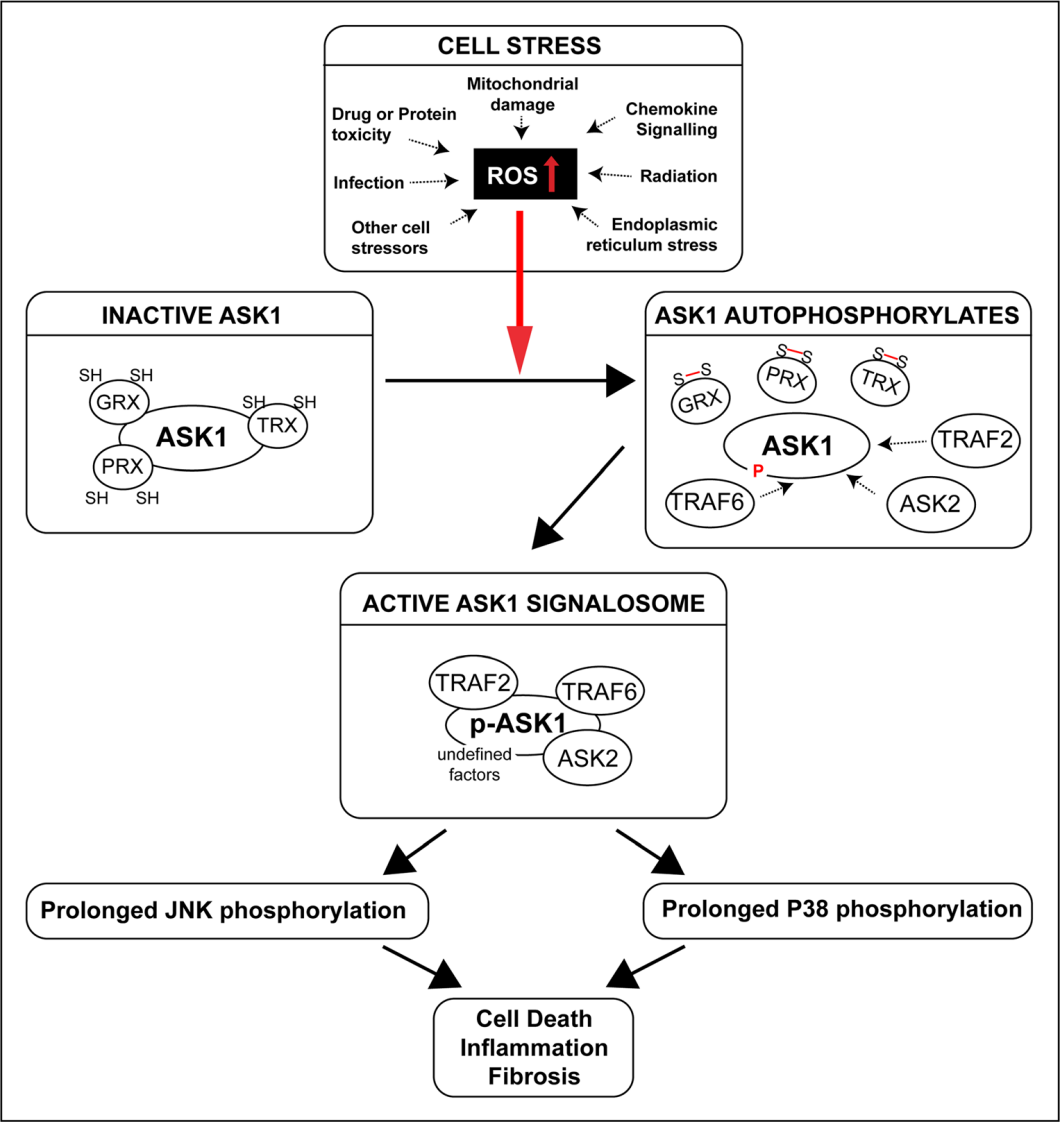
In the redox neutral cell, dithiol oxidoreductases TRX, GRX, and PRX bind to and inactivate ASK1. However, cell stressors can induce cellular oxidative imbalance, which causes disulphide bonds to form between the cysteine residues of TRX, GRX, or PRX. As a result, TRX, PRX, and GRX dissociate from ASK1. Unbound ASK1 is then activated by auto-phosphorylation and a large multicomponent complex forms, referred to as the ASK1 signalosome. The ASK1 signalosome promotes the sustained activation of the P38 and JNK signalling cascades which have been associated with damaging inflammatory responses, cell death, and fibrosis in multiple tissues

The function of ASK1 as an upstream regulator of P38/JNK-mediated disease has received considerable attention in recent literature. ASK1 is widely expressed in many tissues, including the kidney, liver, brain, heart, and lung [49]. Whilst ASK1 is primarily activated in response to oxidative stress, other factors such as calcium overload, endoplasmic reticulum stress, infection, and receptor-mediated inflammatory signals, including lipopolysaccharide (LPS) and tumour necrosis factor (TNF) can also induce ASK1 signalling (reviewed in Shiizaki [28]). As a result, the role of ASK1 has been extensively investigated in disease models, with therapeutic potential observed in diseases of the kidney [50], liver [51], central nervous system [52, 53], joints [54], and cardiovasculature [47, 55, 56]. Subsequently, ASK1 inhibitors have been tested as a therapeutic in various disease settings, ranging from *in vitro* cellular studies to in vivo animal models and large clinical trials (Table 1). Herein, we discuss the potential of ASK1 inhibition for preventing disease, combining evidence from *Ask1* knockout models, and research utilising ASK1 inhibitory compounds.

**Table 1:** A number of ASK1 inhibitors have been developed. The information regarding each is varied. Some have not been extensively studied, whilst others have been used to treat cell lines, small animals, and humans. *NA* indicates information was not available

Inhibitor	Supplier	Notable observations	IC50	Formula	MW	Ref
BPyO-34	Otava Chemicals	NA	520 nM	NA	NA	[57]
GS-444217	Gilead Sciences	ATP competitive inhibitor. Reduced pathological remodelling of the pulmonary vasculature and the right ventricle. Halts progression of pulmonary hypertension in rats. Reduces renal injury in multiple rodent models	2.9 nM	NA	NA	[58–61]
GS-459679	Gilead Sciences	ATP competitive inhibitor. Protects against paracetamol-induced liver injury in mice. Reduces myocardial infarct size and cardiomyocyte apoptosis after acute myocardial ischemia/reperfusion in rats	6.1 nM	NA	NA	[43, 62, 63]
GS-4997 (selonsertib)	Gilead Sciences	ATP competitive inhibitor. Antagonises multidrug resistance in ABCB1- and ABCG2-overexpressing cancer cells. Reduces the progression of liver damage in rodents Phase II clinical trials against diabetes and kidney disease Phase III clinical trials for NASH	3.2 nM	C_24_H_24_FN_7_O	445.502 g/mol	[64–69]
GS-627	Gilead Sciences	Decreased joint damage and inflammation in a rat model of collagen-induced arthritis	4.3 nM	NA	NA	[70]
K811	Kyowa Hakko Kirin Co. Ltd	Nitrogen-containing heterocyclic derivative with high IC50 values for other kinases. Slows disease progression and enhances survival in a mouse model of amyotrophic lateral sclerosis. Limits proliferation of gastric cancer cells and reduces xenograft tumour size in mice	6 nM	NA	NA	[71, 72]
K812	Kyowa Hakko Kirin Co. Ltd	Slows disease progression and enhances survival in a mouse model of amyotrophic lateral sclerosis. Note: lower specificity than K811	NA	NA	NA	[71, 72]
MSC2032964A	Tocris Biosciences	Suppressed autoimmune inflammation in both the spinal cord and optic nerves of a mouse model of autoimmune encephalomyelitis	93 nM	C_16_H_13_F_3_N_6_O	362.31 g/mol	[52]
SRT-015	Seal Rock Therapeutics	Targeted-liver specific ASK1 inhibitor reduces hepatomegaly, fibrosis, and steatosis of the liver	NA	NA	NA	In development
TC ASK 10	Tocris Biosciences	Prevents aberrant smooth muscle growth in patients with chronic obstructive pulmonary disease	14 nM	C_21_H_21_N_5_O.2HCl	432.35 g/mol	[73, 74]

## Kidney disease: ASK1 modulation limits renal inflammation, apoptosis, and fibrosis

Several models of kidney injury indicate that ASK1 activation drives renal damage via the ROS-P38 and JNK signalling cascades [48, 50, 58]. Moreover, studies in animal models of acute kidney injury have shown that *Ask1* knockout or inhibition protects against tubular apoptosis, inflammation, and fibrosis [48, 50]. For example, kidney function, as measured by blood urea nitrogen and serum creatinine levels, is superior in *Ask1*^−/−^ mice following renal ischaemia or unilateral ureteric obstruction when compared to wild-type mice [48, 50]. Lower leukocyte and macrophage infiltration rates also indicate that inflammation is reduced in the damaged kidneys of *Ask1* knockout mice [48, 50]. In rat models of kidney injury induced by auranofin, nephrotoxic serum injection, or unilateral ureteral obstruction, pre-treatment with the ASK1 inhibitor GS-444217 (30 mg/kg via oral gavage) suppresses the ROS-ASK1-P38/JNK response [59, 60]. As a result, kidney function is preserved, due to reduced tubular cell death, inflammation, and fibrosis. Similarly, GS-444217 slows the progression of renal disease in mice with streptozotocin-induced diabetic kidney disease [58]. Streptozotocin treatment damages pancreatic beta cells, causing insulin-dependent diabetes and development of diabetic kidney disease [75]. However, early intervention with GS-444217 reduces renal dysfunction and scarring. Late intervention (up to 15 weeks post diabetes onset) improves renal function and halts the progression of glomerular scarring, tubular injury, and renal inflammation [58]. These observations in the late intervention group suggest that a large treatment window exists for diabetic kidney disease [58]. This study also highlights the tolerability and bioavailability of GS-444217 when delivered in standard mouse chow (0.1% w/w), achieving plasma levels of approximately 20 μmol/L.

The benefits of GS-444217 treatment are even more pronounced in the db/db eNOS^−/−^ mouse model of progressive diabetic kidney disease. Endothelial nitric oxide synthase (eNOS) dysfunction is associated with diabetic nephropathy in humans and eNOS^−/−^ mice have an enhanced susceptibility to kidney damage [76]. When crossed with genetically obese leptin receptor-deficient (db/db) mice, the resultant db/db eNOS^−/−^ progeny robustly model type II diabetic nephropathy. However, daily GS-444217 treatment reduces glomerular scarring, podocyte death, and renal fibrosis, preventing glomerular filtration changes and albuminuria in db/db eNOS^−/−^ mice [59].

Overall, multiple murine models with differing mechanisms of injury have provided evidence that pharmacological inhibition of ASK1 is able to preserve renal function via decreased cell death, inflammation, and fibrosis. These compelling results have subsequently led to a clinical trial testing ASK1 inhibition in humans with diabetic kidney disease (NCT02177786). In 2016, 333 participants took selonsertib (GS-4997) or a placebo, orally, once per day for 48 weeks as part of a phase II clinical trial [64]. The primary outcome measure was a change in glomerular filtration rate (eGFR) from baseline at week 48. This endpoint was not achieved, due to unexpected confounding factors, such as selonsertib-mediated inhibition of creatinine secretion. However, post hoc analysis indicated that the 18 mg selonsertib treatment group had a 71% eGFR reduction and a dose-dependent reduction of p-P38 was noted in all selonsertib treatment groups. This outcome highlights the difficult nature and apparent pitfalls of clinical trial design. However, the results do suggest that selonsertib has a protective effect that warrants further clinical investigation. Therefore, a phase III clinical trial is now underway, to evaluate whether selonsertib can slow the decline of kidney function, reduce the risk of kidney failure, or reduce the risk of death due to kidney disease in participants with diabetic kidney disease (NCT04026165). Notably, these outcome measures are broader than previous selonsertib trials, which will allow for the identification of improvements that may have otherwise been missed using narrower parameters (such as eGFR) to measure kidney function.

## Liver disease: ASK1 modulation prevents hepatocyte death, inflammation, and fibrosis in the liver

Oxidative stress is a primary pathogenic cause of liver disease. For example, excessive ROS production has been associated with alcoholic liver disease, non-alcoholic fatty liver disease, hepatic fibrosis, hepatitis C–induced damage, and poor surgical recovery (reviewed in Lach and Mikalak [77]). Recently, reports of paracetamol-induced, ROS-mediated liver injury have increased significantly, with drug toxicity now a leading cause of acute liver failure [78, 79]. At low doses, paracetamol metabolism generates the reactive metabolite *N*-acetyl-*p*-benzoquinone imine (NAPQI), which is detoxified via glutathione (GSH) conjugation. However, high paracetamol doses cause NAPQI production at a rate exceeding the liver’s detoxification capabilities. NAPQI then forms mitochondrial DNA and protein adducts, inducing oxidative stress [80]. This redox signal causes ASK1-mediated, sustained JNK activation which leads to hepatocyte death [81, 82]. As such, ASK1 inhibition is proposed as a potential therapy for reducing paracetamol-induced liver injury [41].

In a proof of principle study, Nakagawa et al. [41] demonstrated that paracetamol-induced cellular damage in primary hepatocyte cultures was the result of ASK1-mediated JNK activation. Then, studies utilising *Ask1* knockout mice demonstrated that ASK1 deficiency was protective against paracetamol-induced liver toxicity in vivo*.* Notably, the protection conferred in *Ask1* knockout mice was greater than that observed in *Jnk1 and Jnk2* knockout mice, suggesting that compensatory mechanisms may also be blocked by ASK1 inhibition [41]. Overall, three important observations were made regarding ASK1 activation in paracetamol-treated hepatocytes. First, transient P38 and JNK phosphorylation occurred in *Ask1* knockout mice, yet prolonged phosphorylation (3–6 hours post treatment) was significantly reduced. This supports previous reports that ASK1 activation is required for the sustained, pro-apoptotic activation of JNK, whilst the pro-survival role of JNK is not impacted [41]. Secondly, long-term JNK activation was not completely inhibited in *Ask1* knockout mice, illustrating that ASK1-independent mechanisms of JNK activation exist [41]. However, the *partial* reduction of ASK1-mediated JNK1 or JNK2 activation achieved significant liver protection [41]. This suggests that incomplete ASK1 inhibition will be beneficial in vivo, even if functional limitations such as poor drug uptake in a target tissue prevent strong ASK1 inhibition.

The benefit of ASK1 inhibition was then demonstrated in wild-type C57BL/6 mice given hepatotoxic doses of paracetamol. ASK1 inhibition with GS-459679 reduced JNK phosphorylation and centrilobular necrosis [43]. As a result, mRNA levels of inflammatory factors (tumour necrosis factor (TNF-a), interleukin 6 (IL-6), and interleukin 6 1 beta (IL-1β)) remained low and paracetamol-treated mice maintained alanine aminotransferase (ALT) serum levels—which is a strong indicator of liver function [43, 62]. Importantly, GS-459679 was protective when given 90 minutes *after* hepatotoxic paracetamol treatment [62]. This would suggest that a reasonable window of opportunity exists for liver protection in humans experiencing paracetamol overdose, particularly as paracetamol-induced liver damage occurs more slowly in humans than mice [62]. However, this hypothesis remains to be assessed clinically [62].

Beyond paracetamol-induced liver injury, ASK1 deficiency also reduces inflammation and hepatocyte death in a mouse model of obstructive cholestatic injury. In this model, surgical bile duct ligation causes inappropriate bile flow. The subsequent inflammatory response causes fibrosis and cirrhosis of the liver [83]. However, the inflammatory response and overall liver damage induced by bile duct ligation are much lower in *Ask1* knockout mice when compared to controls. For example, infiltrating neutrophil and macrophage activity is reduced in the damaged livers of *Ask1* knockout mice. Notably, *Ask1* knockouts exhibit superior survival when compared to controls receiving the same surgery (78% vs. 51% surviving 21 days post surgery). Overall, serum levels of ALT, aspartate aminotransferase (AST), bilirubin, and bile acid are significantly lower in *Ask1* knockout mice, which also suggests improved liver function when compared to controls [51]. These results indicate that ASK1 inhibition may prevent severe liver damage whilst diagnosis and treatment strategies are implemented in humans with cholestasis.

To date, ASK1 inhibition has predominantly been tested as a therapeutic strategy for limiting non-alcoholic steatohepatitis (NASH). NASH is the most severe form of non-alcoholic fatty liver disease (NAFLD) and the predominant cause of chronic liver failure. NAFLD affects over 25% of adults worldwide and 30% of people with NAFLD will develop NASH [84]. Hepatic oxidative stress, lipotoxicity, and inflammation drive the progression of NAFLD to NASH, causing cirrhosis, fibrosis, or hepatic carcinoma [84]. Currently, there is no approved pharmacological intervention for NASH. Instead, treatment is focused on reducing body weight and increasing physical activity. However, there is a growing need for alternate interventions as the prevalence of obesity and liver disease is increasing [84].

Similar to humans, mice consuming a high-fat diet develop heavy and fibrotic livers, laden with accumulating collagen and triglyceride [85]. However, homozygous *Ask1* knockout mice fed a high-fat diet have healthy livers, comparable to wild-type normal-diet–fed mice [85]. In particular, liver weight and triglyceride content do not increase in *Ask1* knockout mice fed a high-fat diet [85]. ASK1 inhibition also prevents P38 phosphorylation and caspase-3 cleavage in an alternate mouse model of NASH, the nod-like receptor family pyrin domain containing 3 (*Nlrp3*) knock-in transgenic mouse [65]. *Nlrp3* KI mice develop hepatic inflammation and fibrosis due to aberrant NLRP3-mediated inflammation. However, ASK1 inhibition with selonsertib significantly reduces liver fibrosis in *Nlrp3* KI mice (as demonstrated by a 33.5% reduction in hydroxyproline levels when compared to untreated *Nlrp3* KI controls). Moreover, serum ALT levels of untreated *Nlrp3* KI mice are twice as high as the selonsertib-treated *Nlrp3* KI mice, indicating that liver function is retained when selonsertib is used [65]. Selonsertib has now been tested as a treatment for NASH and liver fibrosis in humans.

In 2016, a phase II clinical trial determined that selonsertib was safe and effective in NASH patients with stage 2 or 3 liver fibrosis. In this trial, participants received 24 weeks of open-label, oral selonsertib treatment (6 or 18 mg, daily). Outcomes were assessed by comparing pre- and post-treatment liver biopsies, collagen content, fat content, magnetic resonance elastography, and serum markers of liver injury. Most study participants had stage 3 fibrosis, hepatocellular ballooning, and diabetes mellitus at the beginning of the trial. However, at the trial’s conclusion, the progression of liver disease was notably reduced in selonsertib-treated participants. After 24 weeks of treatment, 13 of 30 individuals in the 18 mg selonsertib group and 8 of 27 in the 6 mg selonsertib group had a one or more stage reduction in fibrosis. Reduced fibrosis was also associated with decreased liver stiffness, collagen content, and lobular inflammation [66, 67]. The observed reduction of fibrosis was particularly exciting, as fibrosis is a strong predictor of liver failure in NASH [67]. Overall, the trial provided evidence that selonsertib treatment is safe and efficacious for individuals with NASH [67]. Selonsertib subsequently progressed to a larger phase III clinical trial for NASH-induced compensated cirrhosis (NCT03053063). This trial was designed to last 240 weeks. However, the trial was discontinued after 48 weeks. The reasoning for this discontinuation is unknown, as the trial results are not yet available. Two other clinical trials utilising selonsertib for NASH treatment are still in progress; a phase II trial for NASH-induced bridging fibrosis (NCT03053050), and a phase II trial combining selonsertib, cilofexor (GS-9674, a non-steroidal FXR agonist), and firsocostat (GS-0976, an ACC inhibitor) for NASH-induced fibrosis (NCT03449446).

## Brain disorders: ASK1 deficiency limits neurodegeneration

Neurodegenerative disorders are a significant and growing health and economic burden. For example, 50 million people globally are living with dementia and this number is expected to increase to 131 million by 2050, due to the ageing population [86, 87]. Of all the neurodegenerative disorders, Alzheimer’s disease is the most prevalent, accounting for approximately 70% of dementia cases. The primary pathological features of Alzheimer’s are the presence of plaques and neurofibrillary tangles in the brain, composed predominantly of beta-amyloid and phosphorylated Tau, respectively. Beta-amyloid impairs mitochondrial function, resulting in excessive ROS production (reviewed in Reddy and Beal [88] and Spuch et al. [89]). An in vitro study of primary neuronal cells derived from embryonic mice suggested that this altered redox state mediates cell death via ASK1-JNK activation [53]. Furthermore, a comparison of neuronal cultures derived from wild-type and homozygote *Ask1* knockout mice indicated that ASK1 deficiency affords remarkable protection against beta-amyloid-induced cell death. After three days of exogenous beta-amyloid treatment, only 20% of the wild-type neurons remained viable compared with 70% of the *Ask1*^−/−^ neurons [53]. In this model, ASK1-JNK activation is the primary effector of neuronal cell death. However, 30% of *Ask1*^−/−^ cells still died, showing that there are ASK1-independent pathways for neuronal cell death [53]. In vivo studies utilising the 5XFAD mouse model of Alzheimer’s disease suggest that P38 activation also mediates beta-amyloid-induced neurodegeneration. 5XFAD transgenic mice express a mutant form of the amyloid precursor protein (APP) containing five familial Alzheimer’s mutations. These mutations have an additive effect, driving beta-amyloid overproduction, and the transgene’s *Thy1* promoter ensures brain-specific expression [90]. As a result, 5XFAD mice develop beta-amyloid plaques, which cause progressive neuronal cell death. By four months of age, 5XFAD mice have significant memory deficits [90, 91]. However, this deficit is reduced in homozygous *Ask1* knockout 5XFAD mice, as measured by the passive avoidance test. P38 phosphorylation is strikingly lower in the 5XFAD/*Ask1*^−/−^ mouse cerebrum, when compared to 5XFAD/*Ask*^*+*/+^ controls. Interestingly, JNK phosphorylation did not change, which contrasts with the JNK activation observed in cultured, beta-amyloid-treated neurons. However, it is likely that the alternate activation of P38 and JNK is due to different *in vitro/in vivo* cell culture conditions. Alternatively, it may indicate that neurodegeneration is driven by more than one apoptotic pathway. Nevertheless, ASK1 deficiency reduced both P38- and JNK-mediated forms of neurodegeneration, which highlights the strength of ASK1 as a common upstream target.

Parkinson’s disease is another common neurodegenerative disease characterised by oxidative stress and activation of the ASK1-P38 and JNK pathways. In vitro, Hu et al. [92] observed significant and persistent ASK1 phosphorylation in dopaminergic neurons (MN9D cell line) treated with the neurotoxic compound oxidopamine. However, targeted ASK1 knockdown, using lentiviral-delivered short hairpin RNA, significantly enhances oxidopamine-treated MN9D survival. In vivo, the same lentiviral shRNA against *Ask1* is protective against oxidopamine toxicity. ShRNA was injected into the mouse substantia nigra pars compacta, reducing ASK1 levels by ~ 80% in tyrosine hydroxylase–positive dopaminergic neurons (as measured by western blot analysis). Three weeks after *Ask1* knockdown, 3 μg of oxidopamine was injected into the left striatum. Seven days after oxidopamine treatment, immunoblotting showed that phosphorylation of both P38 and JNK was significantly reduced in the ASK1-deficient pars compacta. Moreover, histology indicated that twice as many dopaminergic neurons survived in the knockdown group. Behavioural testing, including the corner test and apomorphine-induced rotation test, showed that motor function in the oxidopamine-treated *Ask1* knockdown mice was significantly better when compared to oxidopamine-treated wild-type mice [92]. The benefits afforded by incomplete *Ask1* knockdown (80%) in oxidopamine-treated mice indicate that partial ASK1 inhibition may represent a powerful therapeutic tool, even if complete inhibition is difficult to achieve in a target tissue.

As ASK1-mediated activation of P38 and JNK had been consistently observed in neurons after oxidopamine treatment, in both the MN9D cell line and mice, Hu et al. then examined whether this pathway was active in post-mortem tissue derived from individuals with Parkinson’s disease. Immunofluorescence analysis demonstrated significantly more p-ASK, p-JNK, and p-P38 staining in the Parkinson’s affected tissue compared to matched control samples [92]. Collectively, the observations of p-ASK1, p-P38, and p-JNK in Parkinson’s model systems and human tissue provide strong evidence that ASK1 is a relevant therapeutic target for Parkinsonian disorders.

## Inflammatory disease: ASK1 modulation limits damaging immune responses

Recent studies have associated ASK1-P38 and JNK with the damaging inflammatory responses underlying immune-mediated disease, such as multiple sclerosis, amyotrophic lateral sclerosis, and arthritis [52, 54, 70, 71, 93].

### Neuroinflammation

Multiple sclerosis (MS) is a disease characterised by immune-mediated destruction of the myelin sheath within the central nervous system. In contrast, amyotrophic lateral sclerosis (ALS) is caused by degeneration of the motor neurons within the brain and spinal cord. The aetiopathogeneses of ALS and MS are thought to be quite different; however, the progression of both diseases is characterised by a strong immune response, which may include microglia and astrocyte activation and monocyte or T cell immune responses (reviewed in Zhao et al. [94]).

In a mouse model of MS, where myelin oligodendrocyte glycoprotein (MOG) injections are used to induce T cell–mediated autoimmune encephalomyelitis, ASK1 deficiency affords significant protection against several disease-associated phenotypes. For example, MOG-treated wild-type mice develop significant visual impairment, whereas similarly treated *Ask1* knockout mice do not [52]. Optic nerve histology shows significantly reduced infiltrating inflammatory cells and axonal degeneration in *Ask1*^−/−^ samples [52]. Spinal cord histopathology also indicates that CNS inflammation and glial activation is drastically reduced in MOG-treated *Ask1*^−/−^ mice. In vitro experiments using LPS-treated astrocytes and microglia suggest that the underlying mechanism of MOG treatment is toll-like receptor 4 and 9 (TLR4 and TLR9)–induced P38 signalling. The TLR-P38 pathway also enhanced pro-inflammatory TNFα and nitric oxide synthase activity, which drives demyelination. Both TLR4 and TLR9 are associated with ROS production, providing a logical mechanism for ASK1-mediated P38 activation. The proposed TLR-ROS-ASK1-P38-chemokine pathway to demyelination requires further investigation in vivo. However, chemokine production, inflammation, and demyelination are reduced in MOG-treated *Ask1*^−/−^ mice, supporting the mechanism proposed. Overall, it seems likely that the reduced axonal degeneration and demyelination observed in *Ask1*^−/−^ mice with MOG-mediated autoimmune encephalomyelitis is the result of reduced P38-mediated inflammation [52]. Importantly, ASK1 inhibition (MSC2032964A) achieves similar attenuation of demyelination within the same mouse model and also blocks lipopolysaccharide-binding protein–induced ASK1-P38 activation in cultured mouse astrocytes, providing evidence that ASK1 inhibition may be able to slow the progression of MS [52].

ASK1 deficiency also modulates disease progression in the SOD1 transgenic mouse model of ALS. SOD1 mice carry the familial Cu/Zn-superoxide dismutase (SOD1) mutation, associated with ALS. In this model, aggregates of mutant SOD1 accumulate in the mitochondria causing ROS production, ASK1 phosphorylation, and neuronal cell death mediated by P38 but not JNK [95–97]. *Ask1* knockout does not alter the age of disease onset or prevent ALS lethality in the SOD1 mouse model. However, ASK1 deficiency does mitigate motor neuron death as the disease progresses. As a result, *Ask1*^−/−^ SOD1 mice survive, on average, one month longer than *Ask1*^+/+^ SOD1 mice [98]. ASK1 inhibitors K811 and K812 also reduce ALS pathology in vitro and in vivo. For example, primary spinal cord cultures derived from wild-type mice were infected with lentivirus expressing either a wild-type or mutant form of SOD1. The number of surviving motor neurons in the spinal cord cultures expressing mutant SOD1 was significantly lower than those expressing wild-type SOD1. However, treatment with K811 or K812 completely inhibited mutant SOD1-induced motor neuron death. Similarly, K811 or K812 treatment reduced glial activation and prevented motor neuron death in the SOD1 mouse model. This improved the life expectancy of SOD1 mice by three weeks [71]. The extended lifespan of SOD1 mice treated with ASK1 inhibitors is of particular significance, as riluzole and edaravone, the FDA-approved ALS treatments, do not alter the lifespan of SOD1 transgenic mice [99, 100]. If ASK1 inhibition is able to achieve a similar reduction of motor neuron death in ALS-affected individuals as observed in the ALS mouse model, then the therapeutic effect of ASK1 inhibition would be significantly greater than that of either riluzole or edaravone. However, ASK1 inhibition has not yet been investigated in human tissues derived from individuals with ALS.

### Joint inflammation, arthritis, and bone repair

Rheumatoid arthritis is an autoimmune disease characterised by chronic inflammation, leading to bone and cartilage destruction within the joints. Upregulated P38 activity is a key contributor to this inflammatory response and a number of P38 inhibitors have been tested as potential treatments [6, 54, 101]. However, P38 inhibitors have failed to achieve significant disease attenuation in vivo, possibly because P38 has both pro-and anti-inflammatory effects [11, 24]*.* Alternatively, low doses may have been used to avoid the liver toxicity frequently described when P38 inhibitors are used in vivo, and such doses may be insufficient to achieve meaningful disease attenuation.

Interestingly, experiments utilising synoviocytes derived from individuals with rheumatoid arthritis indicate that JNK activation occurs concurrently with P38 activation and that both must be inhibited to effectively limit inflammatory responses [70]. Therefore, Mninch et al. [54] characterised a mouse model of rheumatoid arthritis to assess the role of ASK1 as an upstream regulator of P38/JNK-mediated inflammation. In this model, serum from arthritic transgenic K/BxN mice is transferred to naive mice. The host’s inflammatory cells, including neutrophils and mast cells, activate in response to the transferred antibodies. This results in elevated levels of inflammatory cytokines, causing significant injury and arthritis within days [102, 103]. However, serum levels of inflammatory cytokines are reduced in treated *Ask1* knockout mice compared to C57BL/6 controls. As a result, *Ask1* knockouts experience significantly less swelling of the joint and reduced cartilage destruction and bone damage when compared to K/BxN-injected wild-type mice [54]. ASK1 inhibition has also been tested in the collagen-induced arthritis rat model [70]. Like the K/BxN serum transfer model, immunisation with type II collagen induces a significant inflammatory response in the host animal, causing arthritis symptoms to manifest within three weeks [104]. However, treatment with the ASK1 inhibitor GS-627 significantly reduces joint damage and swelling in collagen-treated rats [70]. Combined, these two murine models of arthritis suggest that ASK1 inhibition can mitigate the damaging inflammatory response underpinning rheumatoid arthritis.

Whilst rheumatoid arthritis is a systemic inflammatory disease, osteoarthritis tends to manifest as age- or injury-related cartilage destruction. When the joint is initially injured, ROS, cytokines, and growth factors are released into the joint space. As a result, chondrocytes become hypertrophic and proliferative, and the extracellular matrix is degraded. The slow degradation of cartilage increases mechanical stress within the joint, eventually leading to bone remodelling and complete cartilage destruction. Oxidative stress is strongly associated with chondrocyte hypertrophy, mediated by ASK1-P38 and JNK [105, 106]. However, ASK1 inhibition using exogenous thioredoxin blocks the TNFR1–ASK1–P38/JNK signalling pathway in cultured synovial fibroblasts derived from human osteoarthritis knee tissue [93, 106, 107]. In vivo, chondrocyte hypertrophy is significantly reduced in untreated two-year-old *Ask1*^−/−^ mice, when compared to age-matched *Ask1*^+/+^ mice [93]. As a result, *Ask1*^−/−^ mice experience significantly less age-related cartilage degeneration, proteoglycan loss, and calcification of the joints, compared to wild-type controls [93]. Similarly, two surgically induced mouse models of osteoarthritis—one severe (partial meniscectomy) and one mild (joint destabilisation/injury)—suggest that ASK1 deficiency prevents cartilage damage and enhances bone repair [93]. In the severe osteoarthritis model, wild-type mouse joints exhibit extensive cartilage degradation, proteoglycan loss, fibrosis, and inflammatory infiltrates at four weeks post surgery. However, both heterozygous and homozygous *Ask1* knockout mice have significantly reduced bone thinning, cartilage degradation, and fibrosis [93]. Similar results were achieved in the milder joint destabilisation model, with *Ask1*^−/−^ mice showing reduced cartilage hypertrophy and proteoglycan loss eight weeks after surgery, when compared to controls.

Human cartilage collected from individuals with both osteoarthritis and rheumatoid arthritis demonstrate significant ASK1 phosphorylation [70, 93]. This suggests that the benefits of ASK1 deficiency in murine models of arthritis may also be relevant to human disease. Collectively, the research performed to date suggests that ASK1 inhibition is a potential therapeutic strategy to reduce joint degradation in rheumatoid and osteoarthritis. Inflammation and chondrocyte activity are also important for bone formation and repair. Therefore, the observation that ASK1 induces P38/JNK-mediated chondrocyte hypertrophy and death also suggests that ASK1 inhibition may be useful for enhancing fracture repair [93, 107]. Indeed, bone mineralisation and formation is accelerated in *Ask1*^−/−^ mice [107]. However, further studies are required to ascertain the viability of ASK1 inhibition for enhancing human bone repair.

## Cardiopulmonary disease: ASK1 mediates heart and lung phenotypes associated with invasive smooth muscle cells

Smooth muscle cells have an important role for maintaining airway structure and function. However, aberrant smooth muscle activity can cause chronic obstructive pulmonary disease (COPD). In particular, smooth muscle remodelling can impede airway contractility and relaxation, causing thick masses that obstruct airflow (reviewed in Yan et al. [108]). Smooth muscle cells in individuals with COPD show remarkably high p-ASK1 protein levels [73]. Therefore, ASK1 inhibition may represent a therapeutic intervention to limit COPD airway remodelling*.* In vitro studies have shown that ASK1 inhibition, using the compound TC ASK 10 or siRNA *Ask1* knockdown, is able to prevent smooth muscle growth and migration by inhibiting P38 and JNK1 and 2 signalling [73, 74]. The benefits of ASK1 inhibition have not yet been investigated in COPD animal models. Instead, animal studies have focused on the pathological role of ASK1-mediated smooth muscle cell activity during cardiovascular remodelling.

Smooth muscle cells maintain the structural integrity and dynamic properties of blood vessels [109]. However, smooth muscle cells can also cause vascular thickening, known as neointimal hyperplasia after a surgical vascular injury [55]. Similarly, smooth muscle remodelling after myocardial injury can damage the ventricle, causing catastrophic cardiac dysfunction [47]. In both cases, p-ASK1-JNK-mediated apoptosis plays an essential role in the remodelling process and subsequent dysfunction. However, ASK1 inhibition limits pathological vascular remodelling in animal models. For example, when a balloon catheter is inserted into an artery in rats, the intimal endothelial lining is mechanically removed, causing a distending mural injury [110]. Within two minutes of injury, ASK1 phosphorylation occurs, with maximal levels observed five minutes after injury. Notably, ASK1 phosphorylation is elevated sevenfold compared to uninjured controls [55]. ASK1-mediated P38 and JNK activation subsequently promotes vascular smooth muscle cell migration and proliferation. As a result, rats with vascular injury develop neointimal hyperplasia within two weeks [55]. However, pre-treatment infusion of adenovirus vectors expressing a dominant-negative ASK1 mutant (DN-ASK1) in the target artery reduces smooth muscle infiltration and prevents vascular thickening after injury [55]. This outcome has been replicated in mice, with neointimal formation significantly reduced in *Ask1*^−/−^ mice subsequent to an experimental vascular injury induced by cuff placement around the femoral artery [55]. In other mouse models of myocardial infarction or pressure overload-induced cardiac injury, post-injury remodelling impairs cardiac contractility, and changes the ventricular dimensions of wild-type mice [47]. However, *Ask1*^−/−^ mice retain cardiac contractility and exhibit reduced fibrosis compared to wild-type controls [47]. ASK1 inhibition (GS-444217) also prevents pathological vasculature remodelling in two independent rodent models of pulmonary arterial hypertension. In the first model, a single injection of the plant toxin monocrotaline is used to induce pulmonary vascular remodelling. Four weeks after the injection, a peak increase in pulmonary pressure and right ventricle hypertrophy are observed in wild-type rats. However, pulmonary pressure and right ventricle hypertrophy are significantly reduced in mice treated with GS-444217 (0.2% in chow). Importantly, both early (day 7) or late (day 14) interventions are able to significantly improve heart function. Likewise, GS-444217 prevents right ventricle remodelling and pulmonary hypertension in rats injected with the cardiotoxic tyrosine-kinase inhibitor semaxanib and subjected to four weeks of hypoxia [61].

In humans, ASK1 and P38α have also been associated with the pathophysiology of pulmonary arterial hypertension by enhancing the proliferation of fibroblasts and smooth muscle cells [7, 61]. Combined, these findings indicate that ASK1 inhibition may be able to limit pathogenic cardiac or vascular smooth muscle cell remodelling and slow the onset of heart failure. However, a phase II clinical trial, testing selonsertib as a therapeutic for pulmonary arterial hypertension did not achieve its primary endpoint, which was a reduction in baseline pulmonary vascular resistance, as measured by right heart catheterisation [68]. The 24-week randomised trial (NCT02234141) compared three daily doses (2 mg, 6 mg, or 18 mg) of selonsertib to placebo in 150 individuals with pulmonary arterial hypertension. There was a correlation of reduced pulmonary vascular resistance in the higher dose groups (6 mg and 18 mg); however, this was confounded by an increase from baseline vascular resistance in the low-dose group. No treatment groups showed a statistically significant change; however, this may have been due to the large standard deviation observed within each group. For most secondary measures, there was no change from baseline in people who received selonsertib. However, the number of participants that had an improved WHO functional class (a system for defining the severity of an individual’s symptoms and how they impact on day-to-day activities) was notably higher in all selonsertib groups (14–19%) when compared to the placebo group (3%).

## Discussion

The stress-activated MAP kinases P38 and JNK have been associated with apoptosis, inflammation, and fibrosis in multiple disease states affecting the kidney, liver, brain cardiopulmonary systems, and joints. In particular, ROS-mediated, prolonged activation of P38 or JNK is thought to induce damaging cellular responses. In the past, numerous attempts have been made to prevent ROS-mediated disease through the use of antioxidants, small molecule inhibitors, or RNA interference. However, the therapies directed at ROS, P38, or JNK have been disappointing, with a number of limitations being observed. For example, dysregulated ROS production is cytotoxic. However, ROS signalling remains an integral part of normal cellular and physiological function. It is therefore important to target redox stress, without affecting off-target ROS activity. Likewise, P38 and JNK phosphorylation is important for both pro-survival and pro-death signalling, largely dependent on the duration of activity. Therefore, inappropriate inhibition of transient P38/JNK can have critical implications for cellular homeostasis in otherwise normal functioning cells.

In some disease states, P38 and JNK appear to be interchangeably activated, emphasising the potential limitations associated with direct inhibition of either molecule in isolation, whereas ASK1 inhibition has repeatedly been shown to selectively regulate prolonged, apoptotic P38 and JNK activation, but not pro-survival mechanisms [47, 48, 50]. Similarly, ASK1 inhibition is a promising alternative to antioxidant supplementation, as it can reduce the pathological consequences of oxidative stress, without impacting physiological ROS activity.

Many ASK1 inhibitors possessing good potency and selectivity profiles have now been developed (Table 1). These inhibitors are primarily available as small molecules with excellent oral bioavailability and are capable of achieving stable plasma concentrations, with a long half-life and widespread distribution across tissues, including the brain [52, 59, 62, 73]. A number of clinical trials have shown that ASK1 inhibition is well tolerated in humans [64, 66, 68]. There is, however, one major point of concern that is relevant to any therapeutic target within an apoptotic pathway—the potential for aberrant cell survival. For example, reduced ASK1 expression has been associated with aggressive hepatic tumours [111, 112]. Alternatively, enhanced ASK1 expression has a role in the carcinogenesis of human gastric cancer [113]. ASK1 inhibition slows the growth of gastric cancer xenografts in mice and *Ask1*^−/−^ mice have fewer and smaller tumours than wild-type controls in the *N*-methyl-*N*-nitrosourea chemically induced gastric tumourigenesis model [72, 113]. Moreover, platelet-specific deletion of ASK1 prevents tumour metastasis in mice that have established lung cancer, induced by the injection of either melanoma or Lewis lung carcinoma cells [69, 114]. Thus, it is apparent that ASK1 can function as both a tumour suppressor or tumour promoter, depending on the cellular responses induced by ASK1 modulation. It is also worth noting that *Ask1*^−/−^ mice are healthy and long-lived, suggesting that pharmacologically mediated ASK1 inhibition is unlikely to have serious side effects [42, 44–48]. In humans, the ASK1 inhibitor selonsertib has been administered for 12 months in clinical trials, with no increase in malignancies or other significant detrimental effects reported [64]. It seems likely that compensatory cell death mechanisms can prevent neoplastic growth during ASK1 inhibition; however, the potential of carcinogenesis must be considered. It is conceivable however, that individuals with tumours susceptible to ASK1 inhibition could benefit from an adjuvant ASK1 inhibitory therapy that not only enhances the anti-neoplastic effects of chemotherapy, but also affords protection against the frequently observed toxic effects that platinum-based compounds have in the brain, kidney, and ear.

Overall, the evidence available suggests that pro-carcinogenic outcomes resulting from reduced ASK1 activation are unlikely to represent an immediate concern. However, some potential for enhanced bleeding, altered heat production, and oxygen consumption has been observed in *Ask1*^−/−^ mice [115, 116]. These observations suggest that ASK1 inhibition may impact post-operative care and may not be appropriate for individuals with metabolic disease. However, another noteworthy observation is that ASK1 deficiency attenuates lung injury in ventilated mice [117]. Ventilated *Ask1*^−/−^ mice also maintain a more stable heart rate and better oxygen saturation than wild-type controls [117]. Therefore, ASK1 inhibition may provide benefits at the acute and post-ventilation stages of treatment, preventing acute respiratory distress syndrome [117]. Further investigation should determine if similar outcomes are observed in humans. However, it is clear that the potential benefits of ASK1 inhibition will be achieved in a tightly controlled, disease-specific context.

As discussed in this review, ASK1 inhibition has not met defined endpoints in some clinical trials. It is not yet clear what caused these outcomes; however, it may be that ASK1 inhibition had a positive effect that was not captured due to trial methodology. The species effect is another factor, and it is possible that a *Homo sapiens*–tailored approach is required to reap the full benefits of ASK1 inhibition. For this to occur, the publication of full clinical trial results is required. Alternatively, compensatory mechanisms may be at play. For example, numerous studies have shown that ASK1 inhibition significantly reduces unwanted cell death, but in most cases P38 or JNK activity was not completely blocked [50, 53]. At least 14 MKKKs exist that activate the JNK pathway [118], so it is unlikely that ASK1 inhibition is able to completely prevent cell death. Additional MAPKK-independent cell death signals may also be driving apoptosis. Notably, the fundamental interaction of p-ASK1 with specific P38 and JNK isoforms has been limited by a lack of tools for discerning between highly similar proteins. Further investigation will be required to address this issue. Nevertheless, the benefits of ASK1 inhibition or deficiency in pre-clinical disease models are clear. ASK1 does not have to be completely inhibited to have a strong effect and ASK1 inhibition has successfully attenuated disease in models with established tissue damage [58, 62, 92]. Such favourable outcomes suggest that an achievable ‘treatment window’ exists and that ASK1 modulation may benefit human health.

## Summary

ASK1 is a widely expressed and evolutionarily conserved mediator of cell death, fibrosis, and inflammation [49]. The reported beneficial effects of ASK1 deficiency are widespread, indicating that ASK1 inhibition may be a particularly useful therapy in multi-system disorders, such as diabetes-induced tissue damage. Indeed, ASK1 inhibition is beneficial in models of diabetes or high-fat diet–related damage of the cardiovasculature [85, 119], liver [85], kidney [58], and brain [45]. Furthermore, ASK1 inhibition or deficiency has repeatedly limited pathologic activation of P38 or JNK in a disease-specific manner [46–48, 50, 55, 59].

Thus far, the argument for ASK1 inhibition against ROS-P38/JNK-mediated disease is convincing. However, additional disease-specific research is required to fully elucidate the potential benefits of ASK1 inhibition. Within this review, we have illustrated the benefits afforded by ASK1 deficiency in numerous and varied disease models. Many examples now suggest that ASK1 inhibition can limit damaging cell death, inflammation, and fibrosis of the kidney, liver, central nervous system, heart, and lungs. Furthermore, ASK1 inhibition may slow the progression of inflammatory diseases such as multiple sclerosis, arthritis, and amyotrophic lateral sclerosis. Overall, significant protective effects have been observed in numerous tissue types. ASK1 inhibition has great potential and may ultimately be used to achieve lasting, improved human health.

## Footnotes

1 In mammals, four isoforms of P38 have been identified (α, β, γ, and δ) and three isoforms of JNK (1, 2, and 3) (reviewed in [1, 2] respectively). However, the literature does not always specify which specific isoform(s) has been analysed. Throughout this review, specific isoforms are named if they are reported in the primary literature. If isoform-specific information is not available, we have used the general nomenclature P38 or JNK.
